# Chronic kidney disease in Cameroon: a scoping review

**DOI:** 10.1186/s12882-020-02072-5

**Published:** 2020-09-23

**Authors:** Jerry Brown Aseneh, Ben-Lawrence A. Kemah, Stephane Mabouna, Mbeng Emmanuel Njang, Domin Sone Majunda Ekane, Valirie Ndip Agbor

**Affiliations:** 1 Department of Health Research, Health Education and Research Organization (HERO), Buea, Cameroon; 2 Clinical Research Education, Networking and Consultancy (CRENC), Douala, Cameroon; 3 Royal Stoke University Hospital, England, UK; 4 Fundong District Hospital, Fundong, Cameroon; 5 Katholieke Universiteit Leuven, School of Economics and Business, Campus Brussels, Belgium; 6 Nuffield Department of Population Health, University of Oxford, England, UK

**Keywords:** Chronic kidney disease, End stage renal disease, Cameroon

## Abstract

**Objectives:**

This scoping review sought to summarize available data on the prevalence, associated factors, etiology, comorbidities, treatment, cost and mortality of chronic kidney disease (CKD) in Cameroon.

**Methods:**

We searched PubMed, Scopus and African Journals Online from database inception to 31 March, 2020 to identify all studies published on the prevalence, associated factors, etiology, comorbidities, treatment, cost and mortality of CKD in Cameroon.

**Results:**

Thirty studies were included. The prevalence of CKD varied from 3 to 14.1 and 10.0%–14.2% in rural and urban areas, respectively. The prevalence of CKD in patients with hypertension, diabetes mellitus, and human immunodeficiency virus was 12.4–50.0, 18.5%, and 3.0–47.2%, respectively. Hypertension (22.3–59.1%), chronic glomerulonephritis (15.8–56.2%), and diabetes mellitus (15.8–56.2%) were the most common causes of CKD. The cause was unknown in 13.5–17.0% of the cases. Advanced age, hypertension, diabetes mellitus, and obesity were frequent associated factors. Hemodialysis was the main treatment modality in patients with End Stage Renal Disease (ESRD). The monthly cost of management of non-dialyzed CKD was 163 US dollars. The one-year mortality rate of ESRD was 26.8–38.6%.

**Conclusion:**

Chronic kidney disease affects about one in 10 adults in the general population in Cameroon. Patients with hypertension, diabetes mellitus, and human immunodeficiency virus bear the greatest burden of CKD in Cameroon. Advanced age, hypertension, diabetes mellitus, and obesity are major factors associated with CKD. Chronic kidney disease in Cameroon is associated with high morbidity and mortality and huge economic cost on the patient.

## Background

Chronic Kidney Disease (CKD) is an abnormality in kidney structure or function assessed using a matrix of variables including glomerular filtration rate (GFR), thresholds of albuminuria and duration of injury [1]. The global prevalence of CKD in 2015 was estimated at 13.4% [2], with a prevalence as high as 36.1% amongst high-risk populations [3]. Chronic kidney disease poses a serious threat to global health due to its high morbidity and mortality rate [4]. According to the 2015 Global Burden of Disease Study, CKD was the 12th common cause of mortality, accounting for about 1.1 million deaths worldwide [5]. Mortality due to CKD increased by 31.7% over the past decade to represent one of the rapidly rising causes of death worldwide [5]. Chronic kidney disease is the 17th leading cause of global disability-adjusted life years (DALYs) lost to disease [5].

Chronic kidney disease disproportionately affects low-income and middle-income countries (LMICs) with a prevalence that is 15% higher than that in high-income countries [3]. In addition to poorly controlled diabetes mellitus and hypertension, infection, and herbal and environmental toxins play an essential role in the epidemiology of CKD in these settings [6]. Chronic kidney disease is both a cause and consequence of non-communicable diseases (NCDs) [7, 8]. The burden of CKD in LMICs is worsened by limited accessibility to and affordability of renal replacement therapy (RRT) [9]. The number of people requiring RRT worldwide is projected to increase from 3.3 million to 5.4 million people by 2030 with most of this increase in developing countries [10].

High-risk groups for CKD include persons living with hypertension, diabetes mellitus, overweight, obesity [11, 12] and human immune deficiency virus (HIV) [13] as well as the elderly. A meta-analysis conducted in 2018 estimated the pooled prevalence of CKD stages 1–5 and 3–5 in the general African population at 15.8 and 4.6%, respectively [13]. Among high-risk populations, the prevalence of CKD stage 1–5 and 3–5 were 32.3 and 13.3%, respectively [13]. Moreover, the prevalence of CKD was about four times higher in Sub-Sahara Africa compared to North Africa. A large-scale population-based study of about 8000 participants aged 40–60 years from six communities in sub-Saharan Africa revealed an age-standardized prevalence of CKD of 2.4% [14]. By 2030, it is estimated that over 70% of people with end-stage kidney disease will be living in developing countries like countries in sub-Saharan Africa [15] due to the rising prevalence of diabetes mellitus, hypertension, obesity, and HIV in these sub-Saharan countries [16].

The prevalence of CKD in adult Cameroonians varied between 11 and 14.2% [11, 17]. The prevalence of hypertension (31%) [18], diabetes mellitus (6%) [19], and obesity (15%) [20] are high with a prevalence of HIV of 4% [21]. Dialysis was introduced in Cameroon in the early 1980s, and included both peritoneal and hemodialysis [22]. However, hemodialysis has been the only available modality of RRT for over two decades now [22].

This review sought to assess the burden of CKD in Cameroon. Specifically, we summarized data on the prevalence, incidence, risk factors, treatment, cost of treatment, and outcome of patients with CKD in Cameroon. Furthermore, we aimed to describe the economic burden and comorbidities of patients with CKD, and to identify research gaps.

## Methods

This scoping review was conducted according to the approach proposed by Arsksey and O’Malley [23].

### Literature search

PubMed, Scopus and African Journals Online were searched without language restriction to retrieve all publications on the prevalence, the incidence, comorbidity, risk factors, treatment, economic burden and outcome (length of hospital stay and mortality rate) of CKD in Cameroon from database inception to March 31, 2020. Table 1 depicts the search strategy for PubMed which was adapted to suit other databases. The reference list of the selected articles was searched to identify articles which might have been missed during the search.

**Table 1:** Search strategy

Search	Search terms
#1	“Kidney disease*” OR “kidney failure” OR “Renal disease” OR “Renal insufficiency” OR “Chronic kidney” OR “Chronic renal” OR “CKD” OR “CKF” OR “CRD” OR “end-stage renal” OR “end-stage kidney” OR “endstage renal” OR “endstage kidney” OR “uremia” OR “uraemia” OR “dialysis” OR “hemofiltration” OR “haemofiltration” OR “hemodiafiltration” OR “haemodiafiltration” OR “hemodialysis” OR “haemodialysis” OR “renal dialysis”
#2	Cameroon
#3	#1 AND #2
#4	Publication date limits: from database 1 January 1967 to 31 May 2019

### Selection of studies for the review

Cross-sectional, cohort, case-control studies and systematic reviews that reported relevant data on CKD in Cameroon were considered for inclusion. For this review, CKD was defined as estimated glomerular filtration rate < 60 mL/min/1.73m^2^ using either the Modification of Diet in Renal Disease (MDRD) study equation, the Cockcroft-Gault (CG) formula, or the Chronic Kidney Disease Epidemiology Collaboration (CKD-EPI) equations or proteinuria ≥1+ (or albuminuria ≥300 mg/g) or patients with known CKD on RRT [17]. Letters, commentaries, case reports, and case series with less than 30 participants were excluded. For duplicate publications, we considered the most comprehensive or recent report with the largest sample size.

**Fig. 1:**
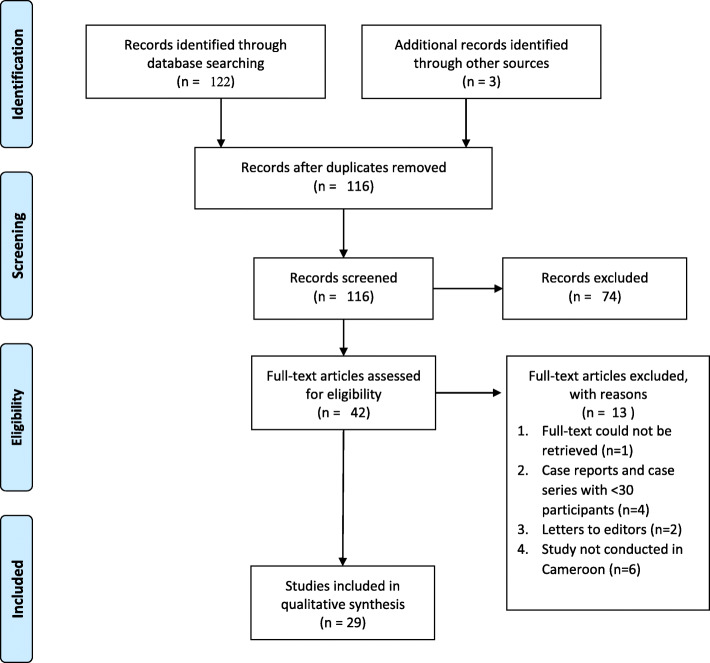
Flow diagram for study screening, selection and inclusion

Two authors independently screened abstracts and citations retrieved from the online search and assessed the full texts of the relevant citations for inclusion in the review, Fig. 1. Disagreements during the study selection process were resolved through consensus, or arbitration by a third review author, in case a consensus could not be reached.

### Data charting

Relevant data were extracted with the aid of pre-structured abstraction sheets. We abstracted the following information from eligible articles: the surname of the first author of the article, publication year, study design and population studied, study setting (community-based or hospital-based), study area (rural or urban), percentage of males included in the study, mean or median age of the participants, sample size, measure used to assess kidney damage or function and if these measures were reassessed after 3 months of measurement, prevalence, comorbidity, treatment rate, median duration of hospital stay and mortality rate of CKD.

## Results

In total, 122 records were retrieved from bibliographic searches. After screening titles and abstracts, 42 full-text papers were assessed for eligibility and 29 studies were retained for this review [11, 17, 24–49].

### Prevalence of CKD in Cameroon

Table 2 summarizes the studies that reported on the prevalence of CKD in Cameroon. The prevalence of CKD was reported in 11 studies in Cameroon [11, 17, 24–32]. All studies were cross-sectional studies, 4 (36.4%) were community-based, and 2 (18.2%) were conducted in rural areas. The average age of the participants ranged from 35 to 61 years.

**Table 2:** Prevalence of CKD in Cameroon

First Author	Year of publication	Study Design	Study Setting	Study area	Disease specific population	Mean Age (in years)	Male (%)	Sample Size	Measure of Kidney damage or Function	Prevalence of CKD
Kaze [24]	2013	Cross-sectional	Hospital-based	Urban	HAART-naïve PLWHA	35.0	32.0%	104	eGFR < 60 based on MDRD and CG or at least 1+ proteinuria	3%
Kaze [17]	2015	Cross-sectional	Community-based	Urban	General adult population	36.5	48.7%	119	eGFR < 60 based on MDRD, CG and CKD-EPI or albuminuria ≥30 mg/g	10.9%
Kaze [17]	2015	Cross-sectional	Community-based	Rural	General adult population	51	39.7	320	eGFR < 60 based on MDRD, CG and CKD-EPI or albuminuria ≥30 mg/g	14.1%
Kaze [11]	2015	Cross-sectional	Community-based	Urban	General adult population	45.3	53.4%	500	eGFR < 60 based on MDRD, CG and CKD-EPI or albuminuria ≥30 mg/g	10.0, 11.0 and 14.2% using CKD-EPI, MDRD and CG, respectively.
Feteh [25]	2016	Cross-sectional	Hospital-based	Urban	Patients with type 2 diabetes mellitus	56.5	53.1%	636	eGFR < 60 based on MDRD	18.5%
Kaze [30]	2016	Cross-sectional	Hospital-based	Urban	Hypertensive adult	60.9	36.6%	336	eGFR < 60 based on MDRD, CG and CKD-EPI or albuminuria ≥30 mg/g	49.7, 50.0 and 52.1% according to MDRD, CKD-EPI and CG equations respectively.
Kamdem [28]	2017	Cross-sectional	Hospital-based	Urban	newly diagnosed and untreated hypertensive patients	51.0	49.1%	839	eGFR < 60 based on MDRD	12.4%
Hamadou [27]	2017	Cross-sectional	Hospital-based	Urban	Hypertensive patients	54.2	33%	400	eGFR < 60 based on CKD-EPI or proteinuria	32.3%
Ekiti [26]	2018	Cross-sectional	Community-based	Rural	Sugarcane plantation workers	39.0	75%	204	eGFR < 60 based on CKD-EPI or at least 1+ proteinuria	3.4%
Halle [32]	2018	Cross-sectional	Hospital-based	Urban	PLWHA attending HIV day clinic	37.1	26.7%	709	eGFR < 60 based on MDRD and CKD-EPI or at least 1+ proteinuria	44% based on CKD-EPI and 47.2% based on MDRD
Kaze [31]	2019	Cross-sectional	Community-based	Urban	General adult population	45.0	48.7%	433	eGFR < 60 based on CKD-EPI or albuminuria > 30 mg/g	11.7%
Temgoua [29]	2019	Cross-sectional	Hospital-based	Urban	First-degree family relatives of HDP	38.3	28.0%	82	eGFR < 60 based on MDRD or at least 1+ proteinuria or diagnosis by a Nephrologist	15.9%

*NR* Not Reported, *NA* Not Available, *HIV* Human immunodeficiency virus, *AIDS* Acquired immune deficiency syndrome, *HAART* Highly active antiretroviral therapy, *PLWHA* Persons living with HIV/AIDS, *OR* odds ratio, *CI* confidence interval, *GFR* Glomerular Filtration Rate, *HDP* Hemodialysis patients, *MDRD* Modification of Diet in Renal Disease, *CG* Cockcroft-Gault, *CKD-EPI* Chronic Kidney Disease Epidemiology

Overall, the prevalence of CKD in the general population ranged from 10.0–14.2%, [11, 17, 31]. The prevalence of CKD ranged from 3.4–14.1% and 10.0–14.2% in the general population in rural [17, 26] and urban areas [17, 31], respectively.

The prevalence of CKD among patients with hypertension ranged from 12.4–52.1% [27, 28, 30], Table 2. Thirty percent of hypertensive patients on treatment in a community-based study were diagnosed with CKD [27], and 12.4% in treatment naïve patients [28]. One study reported a prevalence of CKD of 18.5% among patients with type 2 diabetes mellitus [25]. Two studies evaluated the prevalence of CKD among persons living with HIV/AIDS (PLWHA). The prevalence of CKD in PLWHA ranged from 3.0–47.2% [24, 32].

The prevalence of CKD among sugarcane plantation workers was 3.4% [26]. The prevalence of CKD among first-degree family relatives of persons living with CKD on hemodialysis was 15.9% [29].

### Factors associated with CKD

Table 3 depicts the factors associated with CKD. Advanced age [11, 26–28, 30, 32], female sex [27, 29], obesity/adiposity [17, 27, 30], hyperuricemia/gout [27, 30, 31], longer duration of HIV [32], CD4 count less than 200 cells/mL [32], hyperkalemia [28], dyslipidemia [28, 30], hypertension, diabetes mellitus [11, 17, 30], smoking [17, 30], consumption of alcohol [17, 30] and herbal medication [17], self-medication [30] were associated with increased odds of CKD.

**Table 3:** Factors associated factors of chronic kidney disease in Cameroon

First Author	Year of publication	Study Design	Study Setting	Disease specific population	Mean Age (in years)	Sample Size	Associated Factors (adjusted Odds Ratio; 95% Confidence Interval)
Kaze [17]	2015	Cross-sectional	Community-based	General adult population	47.0	439	History of hypertension (aOR: 3.95; 95% CI, 2.09–7.46),
							History of diabetes mellitus (aOR: 6.64; 95% CI: 2.63–16.75)
							Elevated systolic blood pressure (aOR: 1.01; 95% CI, 1.00–1.02)
Kaze [11]	2015	Cross-sectional	Community-based	General adult population	45.3	500	Advanced age (aOR: 1.09; 95% CI, 1.07–1.12),
							Known hypertension (aOR: 2.40; 95% CI, 1.19–4.82)
							Existing diabetes mellitus (aOR: 3.36; 95% CI, 1.02–11.07),
							Overweight/obesity (aOR: 0.30; 95% CI, 0.17–0.54)
Kaze [30]	2016	Cross-sectional	Hospital-based	Hypertensive adult	60.9	336	Advanced age [aOR: 1.05; 95% CI, 1.02–1.07)
							Raised systolic blood pressure (aOR: 1.01; 95% CI, 1.00–1.02)
Hamadou [27]	2017	Cross-sectional	Hospital-based	Hypertensive patients	54.2	400	Age > 50 years (aOR: 1.75; 95% CI: 1.06–2.89),
							Females (aOR: 2.21; 95% CI: 1.29–3.78), obesity (aOR: 1.58; 95% CI: 1.36–1.95), hyperuricemia (aOR: 3.67; 95% CI: 1.78–7.58)
Kamdem [28]	2017	Cross-sectional	Hospital-based	newly diagnosed and untreated hypertensive patients	51.0	839	Age > 55 years (aOR: 5.29; 95% CI, 3.33–8.42), obesity (aOR: 0.15; 95% CI, 0.10–0.26), hyperkalemia (aOR: 1.33; 95% CI, 1.03–1.72)
Ekiti [26]	2018	Cross-sectional	Community-based	Sugarcane plantation workers	39.0	204	Age ≥ 40 years (aOR: 18.7; 95% CI: 1.5–236.4)
Halle [32]	2018	Cross-sectional	Hospital-based	PLWHA attending HIV day clinic	37.1	709	age > 35 years (aOR: 1.04; 95% CI: 1.02 to 1.06), longer duration of HIV (aOR: 2.60; 95% CI: 1.53 to 3.95), history of Hepatitis B (aOR: 3.04; 95% CI, 1.08 to 8.54),
							CD4 count less than 200 cells/mL (aOR: 3.64; 95% CI, 2.55 to 5.21)
Kaze [31]	2019	Cross-sectional	Community-based	General adult population	45.0	433	Increased systolic blood pressure (aOR: 1.02; 95% CI, 1.00–1.04) per mmHg higher SBP), hyperglycemia (aOR: 4.73; 95% CI, 1.24–18.08) and hyperuricemia (aOR: 3.12; 95% CI, 1.58–6.16)

*HIV* Human immunodeficiency virus, *AIDS* Acquired immune deficiency syndrome, *HAART* Highly active antiretroviral therapy, *PLWHA* Persons living with HIV/AIDS, *aOR* adjusted odds ratio, *CI* confidence interval, *GFR* Glomerular Filtration Rate, *CKD-EPI* Chronic Kidney Disease Epidemiology, *SBP* systolic blood pressure

### Etiologies of chronic kidney disease in Cameroon

Eight studies reported on the etiologies of CKD in Cameroon, Table 4. Overall, hypertension (22.3–59.1%), chronic glomerulonephritis (15.8–56.2%), diabetes mellitus (7.3–24.0%) and HIV (6.6–11.5%) were the main etiological factors of CKD. The etiology of CKD was unknown in 13.5–17.0% of cases [35–42]. Halle et al 2016 reported hypertension (30.9%), glomerulonephritis (15.8%), diabetes mellitus (15.9%) and HIV (6.6%) as the major etiologies of CKD in a chart review of 863 medical records [37]. In a prospective study of 661 patients, the major etiologies of CKD were hypertension (28.3%), chronic glomerulonephritis (17.5%), diabetes mellitus (13.9%) and HIV (6.7%) [39].

**Table 4:** Etiology of CKD in Cameroon

First author	Year of publication	Study area	Study Design	Study setting	Study population	Mean age (in years)	Male (%)	Sample size	Etiologies
Halle [35]	2014	Urban	Cross-sectional	Hospital-based	Patients on maintenance hemodialysis	49.4	66.4	113	Hypertension (25.6%), Chronic glomerulonephritis (20.6%), diabetes mellitus (17.4%)
Kaze [36]	2014	Urban	Cross-sectional	Hospital-based	Patients on maintenance hemodialysis	52.7	64.0	45	Hypertension (29%), chronic glomerulonephritis (24%), Diabetes mellitus (24%)
Halle [37]	2015	Urban	Retrospective cohort	Hospital-based	Patients with ESRD	47.4	66.0	863	Hypertension (30.9%), glomerulonephritis (15.8%), diabetes mellitus (15.9%), HIV (6.6%), unknown (14.7%)
Kaze [38]	2015	Urban	Retrospective cohort	Hospital-based	Patients admitted in the nephrology unit	44.8	60.0	225	Chronic glomerulonephritis (25.9%), hypertension (22.3%), diabetes mellitus (20.1%)
Halle [39]	2016	Urban	Prospective cohort	Hospital-based	Patients on maintenance hemodialysis	46.3	66.0	661	Hypertension (28.3%), chronic glomerulonephritis (17.5%), diabetes mellitus (13.9%), hypertension and diabetes (7.3%), HIV (6.7%), unknown (16.9%)
Halle [40]	2016	Urban	Cross-sectional	Hospital-based	Maintenance hemodialysis	51	66.0	97	Hypertension (25.8%) Chronic glomerulonephritis (20.6%) Diabetes mellitus (17,5%)
Luma [41]	2017	Semi-urban	Cross-sectional	Hospital-based	Hemodialysis patients	48	65.4	104	Hypertension (40.4%), chronic glomerulonephritis (19.2%), HIVAN (11.5%), Diabetes mellitus (7.7%), obstructive nephropathy (2.9%), unknown (13.5%)
Moor [42]	2017	Urban	Cross-sectional	Hospital-based	Patients on maintenance hemodialysis	55	75.0	44	Hypertension (59.1%), Diabetes mellitus (11.4%)

*NR* Not Reported, *ESRD* End stage renal disease, *HIVAN* HIV associated nephropathy

### Major comorbidities in CKD patients in Cameroon

Thirteen studies discussed the comorbidities of CKD in Cameroon, Table 5. Ten or more of these studies reported hypertension (25.8–95.6%) and diabetes mellitus (11.40–41.54%)as major comorbidities associated with CKD patients. Also, viral infections such as HIV (4.4–13.5%), Hepatitis B (6.2–12.6%) and Hepatitis C (19.2–26.8%) infections were also important comorbidities associated with CKD. Furthermore, hyperuricemia, obesity, previous cardiovascular events, malnutrition, anemia, smoking, and alcohol use were major comorbidities.

**Table 5:** Major comorbidities in Chronic Kidney Disease patients in Cameroon

First author	Year of publication	Study area	Study population	Mean age (in years)	Sample size	Comorbidities
Halle [43]	2009	Urban	Patients with CKD	50.1	140	Hypertension (62.1%); diabetes mellitus (25.0%); gout (7.1%); HIV (6.4%)
Halle [35]	2014	Urban	ESRD patients on dialysis	49.4	113	Mid-arm muscle circumference (23.9%); heart failure (22.1%); diabetes mellitus (20.3%); HIV (4.4%)
Kaze [36]	2014	Urban	Patients on maintenance hemodialysis	52.7	45	Hypertension (95.6%); anemia (42%); left ventricular hypertrophy (60%); valvular heart disease (51.1%); heart failure (33.3%); dyslipidemia (33.3%); diabetes mellitus (24%); tobacco use (22.2%); obesity (4%)
Kaze [38]	2015	Urban	Patients with CKD	44.8	139	Hypertension (81.3%); diabetes mellitus (32.2%); tobacco use (15.1%); HIV (10.1%)
Mbouemboue [44]	2016	Semi-urban	ESRD	45.0	35	Anemia (Females [100%]; Males [92%])
Halle [40]	2016	Urban	Maintenance hemodialysis	51.0	97	Hypertension (25.8%); Diabetes mellitus (17.5%); HCV (20.6%); HIV (8.2%); HBV (6.2%)
Kouotou [45]	2016	Urban	Hemodialyzed patients	48.6	112	Hypertension (66.1%); Diabetes mellitus (25.9%); HCV (26.8%)
Hamadou [27]	2017	Urban	Patients diagnosed with CKD	54.2	400	Anemia (44.5%), Obesity (39.75%), Diabetes mellitus (32%); hyperuricemia (10.75%); tobacco use (0.8%)
Moor [42]	2017	Urban	Patients on maintenance hemodialysis	55.0	44	Hypertension (59.1%); Diabetes mellitus (11.4%); alcohol use (11.4%); tobacco use (4.5%)
Luma [41]	2017	Semi-urban	Patients on maintenance hemodialysis	48.0	104	Hypertension (84.6%); HCV (19.2%); HIV (13.5%); HBV (10.6%)
Lemogoum [46]	2018	Urban	Patients with CKD	52.0	150	Hypertension (87.3%); dyslipidemia (62.0%); overweight/obesity (53.3%); abdominal obesity (34.0%); Diabetes mellitus (32.7%); previous cardiovascular event (18.0%)
Doualla [47]	2018	Urban	Non-dialysed CKD patients	55.8	103	Hypertension (87.4%); Diabetes mellitus (34.0%); gout (21.4%); HIV (12.6%)
Halle [34]	2019	Urban	Patients with CKD	53.1	130	Hypertension (70.77%); diabetes mellitus (41.54%); HIV (8.5%); gout (6.9%)

*CKD* Chronic kidney disease, *ESRD* End-stage renal disease, *CRF* Chronic renal failure, *HIV* Human immunodeficiency syndrome, *HBV* Hepatitis B, *HCV* Hepatitis C

### Treatment of CKD in Cameroon

Most of the CKD patients required hospitalization and eventual dialysis. The hospitalization rate was 42.2% in patients who were referred late to the nephrologist, and 33.6% of these late referrals were proposed emergency dialysis [43]. Emergency unplanned dialysis on a temporary catheter was required in 88.3% of 863 adult patients with CKD [37].

### Cost of CKD management in Cameroon

Data on the economic burden of CKD is scarce in Cameroon. In a one-month retrospective cost analysis of non-dialysis CKD stage 3–5 patients, the total cost for management of CKD was 163 USD, 86.4% of which was from direct medical cost [33]. Meanwhile, only 1.4% of the 69 participants, with a median monthly salary of 162 USD, had full health insurance coverage [33].

### Mortality of CKD in Cameroon

The mortality rate of CKD in Cameroon ranged between 26.8 and 58.0% during a period of 1 to 10 years of follow up, Table 6 [39, 48, 49]. An audit of 661 medical records reported a 10-year mortality rate of 44.9% [39]. The highest mortality rate of 58.0% was reported in a 15 months’ prospective study in 197 ESRD patients. Furthermore, the one-year mortality rate of hemodialyzed patients in a retrospective study was 29.8% [49]

**Table 6:** Mortality of CKD in Cameroon

First author, publication year	Study area	Study Design	Study setting	Study population	Median age	Sample size	Mortality rate
Halle 2016 [41]	Urban	Retrospective cohort	Hospital-based	ESRD patients on hemodialysis	46.3	661	12-month mortality = 26.8% 10-year mortality = 44.9%
Fouda 2017 [48]	Urban	Prospective cohort	Hospital-based	ESRD patients on dialysis	48.0	197	15-month mortality = 58.0%
Halle 2018 [43]	Urban	Retrospective cohort	Hospital-based	PLHIV with ESRD on hemodialysis	46.0	57	12-month mortality = 38.6%

*NR* Not Reported, *ESRD* End-stage renal disease, *PLHIV* People living with Human Immunodeficiency Virus

## Discusssion

This scoping review systematically summarizes data on the prevalence, associated factors, etiology, comorbidities, treatment and its cost, and mortality of CKD in Cameroon. The prevalence of CKD was high, ranging from about 1 in every 10 people in the general population to about 1 in every 2 persons in high-risk groups. Hypertension, diabetes mellitus and chronic glomerulonephritis were the most common causes of CKD, while the cause was unknown in a significant proportion of patients. Hypertension, diabetes mellitus, obesity, advanced age and female gender were some factors associated with developing CKD in Cameroon. The treatment of CKD involved the management of comorbidities, progression factors, and hemodialysis in those with ESRD. Despite these treatment measures, mortality from CKD remains high with a 1-year mortality rate of more than 25% among hemodialyzed patients. The cost of non-dialysis treatment was high relative to the monthly income of patients with CKD.

The prevalence of CKD was reported in both the general population and in high-risk populations (persons with hypertension, diabetes mellitus, obesity, and HIV) applying various estimators of GFR. The prevalence of CKD in the general population ranged from 10 to 14.2%, which is similar to the overall prevalence of 15.8% in the African adult population [13]. In rural areas, the prevalence was higher compared to urban areas which was similar to the findings of Stanifer and colleagues [15]. This could be due to the low awareness of CKD risk factors such as consumption of nephrotoxic herbal concoctions and alcohol in rural settings. Compared to the general population, the prevalence of CKD was higher in high-risk populations, which was similar to the findings of Kaze and colleagues [13].

About a third to half of patients with hypertension in Cameroon had CKD [27], this was higher than the prevalence reported by Bahrey and colleagues (about 1 in 5 hypertensives had CKD) in Ethiopia [50]. This discrepancy might be due to differences in the characteristics of the study population. The prevalence of CKD in newly diagnosed patients with hypertension in Cameroon was 12.4% [28], which was much lower than the prevalence among known hypertensives on treatment (32.3%) [27] and those not on treatment (52.1%) [30]. Compared to treated hypertensive patients, those newly diagnosed with hypertension are more likely to have lived with the disease for a shorter duration; and as a result, are less likely to develop CKD. The prevalence of CKD in PLWHA was 3%, which was comparable to the findings of Kabore and colleagues [51]. We observed a prevalence of CKD among persons living with diabetes mellitus of 18.7%. In a systematic review in Africa, the prevalence of CKD among patients with diabetes was found to vary widely between 11 and 83.7% [52]. The duration of diagnosis and comorbidities played a significant role on the prevalence of CKD among patients with diabetes mellitus [52].

Advanced age and hypertension were common factors associated with CKD in Cameroon and this was similar to other African settings [14, 53]. Overweight and diabetes were independently associated with CKD, which is in line with the findings of Bahrey and colleagues [50]. Females were more likely to develop CKD, and this was coherent with the findings in a study in Uganda [54]. Hypertension, diabetes mellitus, and chronic glomerulonephritis were identified as the most common causes of CKD in our study. No cause for CKD was identified in about 15% of cases.

The most common comorbidities among patients with CKD in our review were hypertension, diabetes mellitus, anemia, obesity, and cardiac diseases. This was similar to the findings of Fraser and colleagues who highlighted hypertension, diabetes mellitus, anemia and ischemic heart disease as common comorbidities associated with CKD [55]. Whether hypertension is a consequence or an etiology of CKD, depends on which was diagnosed first as hypertension is an established cause and consequence of CKD. Anemia is almost always associated in the course of kidney disease because of the kidney’s role in erythropoiesis. Cardiovascular diseases are established comorbidities of CKD likely due to other cardiovascular risk factors such as hypertension and diabetes mellitus in these patients. Cardiovascular diseases were twice more common among CKD patients compared to the general population and advances at twice the rate [56]. Additionally, hyperuricemia was identified in non-dialyzed CKD patients followed in referral centers and as a factor of progression of CKD [47].

Hepatitis B virus, hepatitis C virus, and HIV were found to be common comorbidities in hemodialysed CKD patients [40, 41]. The hemodialysis procedure raises an issue of safety coupled with disturbances in both innate and adaptive immunity in those on maintenance hemodialysis. Hence, rendering these patients more susceptible to these blood-borne viral infections.

The growing burden of CKD is paralleled by the need to curtail those who end up in ESRD requiring renal replacement therapy (RRT). Effective and practical therapies for CKD remain a challenge even in developed countries [57]. Little is known about the cost of management of CKD in Cameroon. Nevertheless, it is estimated that these patients have to pay about 12 US Dollars per dialysis session [22]. The management of CKD was costly, especially in a population with low health insurance coverage as discussed in another study in non-dialysed stage 3–5 CKD patients [33]. In the USA, the cost of medical care of CKD patients even doubled when there were comorbid conditions [58]. Chronic kidney disease is associated with a huge economic burden in low-income settings, and limited access to treatment centers, essentially hemodialysis centers that are located sparingly in urban areas.

Chronic kidney disease has a high mortality rate among patients with ESRD. Over a quarter of patients starting hemodialysis die within the first year, with about half within the first six months. Chronic kidney disease patients with co-existing hypertension and diabetes mellitus conveyed the poorest prognosis. Late presentation of CKD and affordability were cited as major drivers of high early mortality [39]. Slowing the progression of CKD to ESRD is significantly hampered in our setting by the late presentation of these patients at nephrology centres. In developed settings, there are more effective referral strategies to nephrologists for a prompt management of CKD, and CKD-related complications or comorbidities [59]. Instituting a screening program and national CKD registry, and improving the availability, accessibility, and affordability of dialysis care in Cameroon are crucial to reducing the burden of CKD in Cameroon.

Our review had some key limitations. The prevalence of CKD reported by studies using a single time-point assessment of kidney function or damage are likely to be biased estimates of the true prevalence. Since serum creatinine has a high intraperson variability, a single time point measurement would lead to random misclassification of participants as cases or non-cases of CKD. This error is worse in small studies. Having a large enough sample size with control measurement of serum creatinine levels after three months is important to account for this random error by regression to the mean. The fact that the formulae used to estimate glomerular filtration rate have not been validated in the African population further complicates efforts to estimate the incidence and prevalence of CKD in this population. In addition, limited financial and human resources are major barriers to ascertain the diagnosis of CKD in epidemiological studies, especially in Cameroon. There was a substantial degree of heterogeneity across study participants in the studies included in this review. Studies reporting on the causes of CKD were cross-sectional, which do not provide evidence of temporality. Therefore, it is impossible to know if, for example, hypertension in a given patient is a cause or consequence of CKD. These limitations highlight the need for collaborative efforts to better understand the epidemiological profile of CKD in Cameroon.

## Conclusion

Chronic kidney disease represents a significant cause of morbidity and mortality in Cameroon. The prevalence of CKD was highest among patients with hypertension, diabetes mellitus, and HIV. The main causes include hypertension, diabetes mellitus, chronic glomerulonephritis, HIV and unknown in some cases. Potential actions to curb the burden of CKD in Cameroon include raising public awareness about the disease, encouraging timely referral from general practitioners to nephrologists, increasing the availability of treatment centers, and encouraging health insurance to cover some of the cost of care.

### Research perspective

There is limited data on the incidence and prevalence of CKD in the general population. Factors associated with CKD has been generated mostly from cross-sectional studies with possibility of reverse causation. There is a need for population-based cohort studies to assess the incidence and risk factors of CKD in Cameroon. A less costly approach to assess the risk factors of CKD would be to conduct a case-control study using population-based controls. In addition, more research is needed to assess the mortality rate of CKD and its predictors in patients with ESRD. Studies evaluating the economic burden of CKD in Cameroon are warranted. Creation of a national registry for CKD patients may help foster research in CKD in Cameroon and improve on its management and survival rate.

## Data Availability

Available data can be obtained by contacting the corresponding author.

## Abbreviations

CKD: Chronic Kidney Disease
DALYs: Disability-adjusted life years
ESRD: End Stage Renal Disease
GFR: Glomerular Filtration Rate
HDP: Hemodialysis patient
HAART: Highly active anti-retroviral therapy
NCD: Non-communicable disease
PLWHA: People living with HIV/AIDS
RRT: Renal Replacement Therapy
